# Supplementary material for: Genome-wide CRISPR Screens in T Helper Cells Reveal Pervasive Crosstalk between Activation and Differentiation
Source: Cell. 2019 Feb 7;176(4):882–896.e18. doi: 10.1016/j.cell.2018.11.044 (PMC6370901; doi:10.1016/j.cell.2018.11.044)
Supplement: Data S2. Processed Data from All the Steps of the Analysis, Related to Figure 1 [file mmc2.zip › supplemental data/motif analysis/Stat6_SRX021632_homer/homerResults/motif16.similar.html]

motif16

## Information for motif16

A
C
T
G
A
T
C
G
C
T
G
A
C
G
T
A
G
C
T
A
C
G
A
T
G
A
T
C
G
T
A
C
A
G
T
C
A
T
G
C
  
Reverse Opposite:  

T
A
C
G
A
C
T
G
C
A
T
G
C
T
A
G
G
C
T
A
C
G
A
T
C
G
A
T
A
G
C
T
A
T
G
C
T
G
A
C
  

|  |  |
| --- | --- |
| p-value: | 1e-86 |
| log p-value: | -1.998e+02 |
| Information Content per bp: | 1.709 |
| Number of Target Sequences with motif | 564.0 |
| Percentage of Target Sequences with motif | 2.23% |
| Number of Background Sequences with motif | 206.7 |
| Percentage of Background Sequences with motif | 0.86% |
| Average Position of motif in Targets | 93.8 +/- 52.2bp |
| Average Position of motif in Background | 103.4 +/- 56.7bp |
| Strand Bias (log2 ratio + to - strand density) | -0.2 |
| Multiplicity (# of sites on avg that occur together) | 1.02 |
| Motif File: | file (matrix) reverse opposite |

### Similar de novo motifs found

|  |  |  |  |  |  |  |  |
| --- | --- | --- | --- | --- | --- | --- | --- |
| Rank | Match Score | Redundant Motif | P-value | log P-value | % of Targets | % of Background | Motif file |
